# Nonhemolysis of epidemic El Tor biotype strains of *Vibrio cholerae* is related to multiple functional deficiencies of hemolysin A

**DOI:** 10.1186/s13099-019-0316-7

**Published:** 2019-07-12

**Authors:** Yufeng Fan, Zhenpeng Li, Zhe Li, Xu Li, Huihui Sun, Jie Li, Xin Lu, Weili Liang, Biao Kan

**Affiliations:** 1 State Key Laboratory of Infectious Disease Prevention and Control, National Institute for Communicable Disease Control and Prevention, Chinese Center for Disease Control and Prevention, No. 155, Changbai Road, Changping, Beijing, 102206 China; 2 Collaborative Innovation Center for Diagnosis and Treatment of Infectious Diseases, Hangzhou, China

**Keywords:** *Vibrio cholerae*, *hlyA*, Genotype, Hemolysis

## Abstract

**Background:**

Hemolysis of bacteria is an important phenotype used for typing and characterizing strains with specific biomarkers and even a virulence factor in bacterial pathogenesis. In *Vibrio cholerae*, hemolysin HlyA is responsible for hemolysis of sheep red blood cells, and this hemolytic phenotype is used as a biotyping indicator and considered one of the virulence factors. At the beginning of the seventh cholera pandemic, the El Tor biotype strains of serogroup O1 were distinguished by hemolysis from the sixth pandemic O1 classical biotype strains, whereas during the following epidemics, nonhemolytic El Tor strains appeared, suggesting phenotypic and genetic variations in these strains. This study aimed to investigate the possible mechanisms involved in nonhemolysis of El Tor strains.

**Results:**

Five sequence types of *hlyA* genes were found in the studied O1 El Tor strains isolated during the seventh pandemic. A 4-base deletion in *hlyA* caused the HlyA protein mutation and non-hemolytic phenotype. Some strains carry wildtype *hlyA* genes but are still non-hemolytic, and greatly reduced *hlyA* transcription and blocked secretion of hemolysin were observed in hemolysis tests of the subcellular components and transcription/expression analysis of *hlyA*.

**Conclusions:**

Mechanisms responsible for nonhemolysis of the epidemic O1 El Tor strains are complex and not only confined to gene mutation but also deficiencies of transcription and extracellular transport of HlyA. Mutations in gene regulation and protein secretion systems of HlyA in the nonhemolytic *V. cholerae* strains should be areas of concern in future studies.

**Electronic supplementary material:**

The online version of this article (10.1186/s13099-019-0316-7) contains supplementary material, which is available to authorized users.

## Background

The seventh cholera pandemic began in 1961, and it is still considered pandemic at present. Among nearly 210 serogroups of *Vibrio cholerae*, only the toxigenic serogroups O1 and O139 cause cholera epidemics. The causal strains of the seventh pandemic belong to serogroup O1, El Tor biotype, which can generate multiple toxins (mainly cholera toxin, CT) and toxin-related factors, and it can trigger severe watery diarrhea, dehydration and other clinical manifestations. Furthermore, the toxin-coregulated pilus (TCP), which promotes the colonization of *V. cholerae* in the human small intestine [1], and neuraminidase (NANase), which modifies and promotes the binding of CT to GM_1_ ganglioside in small intestinal epithelial cells [2], are considered important pathogenic factors. Additionally, hemolysin is also recognized as an exogenous toxin secreted by *V. cholerae* [3, 4].

Hemolysin is an important virulence factor in many pathogenic bacteria, such as *Streptococcus suis* [5], *Listeria monocytogenes* [6], *V. parahaemolyticus* [7] and others. These hemolysins damage cells by forming pores in the cell membrane. In *V. cholerae*, hemolysin (HlyA) is encoded by the gene *hlyA*, which is located on *V. cholerae* chromosome II and the product of which is secreted via the Type I Secretory System (T1SS) [8]. HlyA has been demonstrated to exert hemolytic activity, lethality and cardiotoxicity in *V. cholerae*, especially in some nontoxigenic non-O1/non-O139 serogroups [9, 10]. It not only dissolves red blood cells and other cells, but it also triggers apoptotic cell death during infection [11]. Hemolysin acts on the target cell membrane, inserting into the lipid bilayer and forming a pentamer channel [12], which causes a large number of intracellular components to leak out and leads to cell death.

Hemolytic phenotypes of bacteria are also used for biological typing of bacteria. Among the traditional biological typing of pathogens such as *S. suis* [13] and *Staphylococcus*, the hemolytic phenotype is used as one of the phenotypic typing tests. Historically, hemolysis of *V. cholerae* has been used as a biological test to distinguish classical and El Tor biotypes of serogroup O1 [14]. However, a large number of non-hemolytic El Tor strains of *V. cholerae* later emerged in the seventh pandemic, revealing the genetic variation among the newly epidemic El Tor strains. Therefore, hemolysis is no longer used to identify these two biotypes [15]. In the 1970s, a phage-biotyping scheme was established in China for the biological subtyping of the epidemic El Tor strains [15]. In this scheme, the sensitivities to the typing bacteriophages and the biological tests, including sorbitol fermentation and hemolysis, were used to classify the tested El Tor strains into different phage-biotypes [16], a technique that is applied for the subtyping of O1 El Tor strains and in the surveillance of cholera. Hemolysis-positive and negative El Tor strains are found in different epidemic years.

Genomics analysis has revealed that seventh pandemic strains of *V. cholerae* are highly clonal, characterized by individual, genetically monomorphic lineages, with successive accumulation of mutations during the process of spreading [17, 18]. However, hemolytic and nonhemolytic strains were observed in different cholera epidemics. The phenotypic difference may be caused by genetic variance, whereas it is not clear whether these opposite phenotypes resulted from the presence/absence of hemolysin genes, mutations in hemolysin genes, or the expression of these genes. Therefore, analyses of the determinants of hemolysis and nonhemolysis variance are conducive to the discovery of genetic mutations in these high clonal strains.

In this study, we focused on the hemolysis phenotype of the El Tor strains isolated in the seventh pandemic, with the goal of analyzing the hemolysin gene variance and the activities of the hemolysin in these hemolytic and nonhemolytic El Tor strains of *V. cholerae*. We found that in addition to *hlyA* gene mutation, deficiencies in the expression and transport of HlyA may also have the roles to nonhemolysis of the strains.

## Results

### Variance of *hlyA* genes among the hemolytic/nonhemolytic strains

**Fig. 1:**
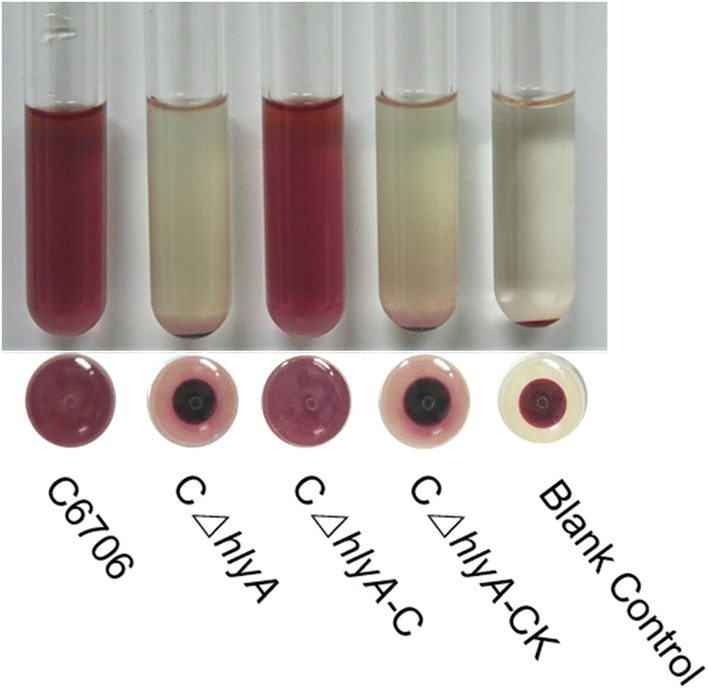
Identification of the role of the *hlyA* gene in hemolysis of strain C6706. Deletion of *hlyA* from C6706 caused a negative result for strain CΔ*hlyA* in the hemolysis test, and a positive was obtained when *hlyA* gene was complemented (CΔ*hlyA*-C). Strain CΔ*hlyA* complemented with the vector pBAD33 was used as the negative control, and 1% sheep erythrocyte LB solution was used as the blank control

C6706 is a strain of *V. cholerae* El Tor biotype in the seventh pandemic with a typical hemolytic positive phenomenon and intact *hlyA* gene. We constructed a *hlyA* deletion strain from C6706, named CΔ*hlyA*, and a *hlyA* complemented strain, CΔ*hlyA*-C, by transformation of the recombinant plasmid pBAD33-*hlyA* carrying intact *hlyA* into CΔ*hlyA*. The hemolysis tests showed that CΔ*hlyA* lost the hemolysis phenomenon, but CΔ*hlyA*-C regained hemolytic capacity (Fig. 1), showing that HlyA contributed mainly to hemolysis of the epidemic El Tor strain.

**Fig. 2:**
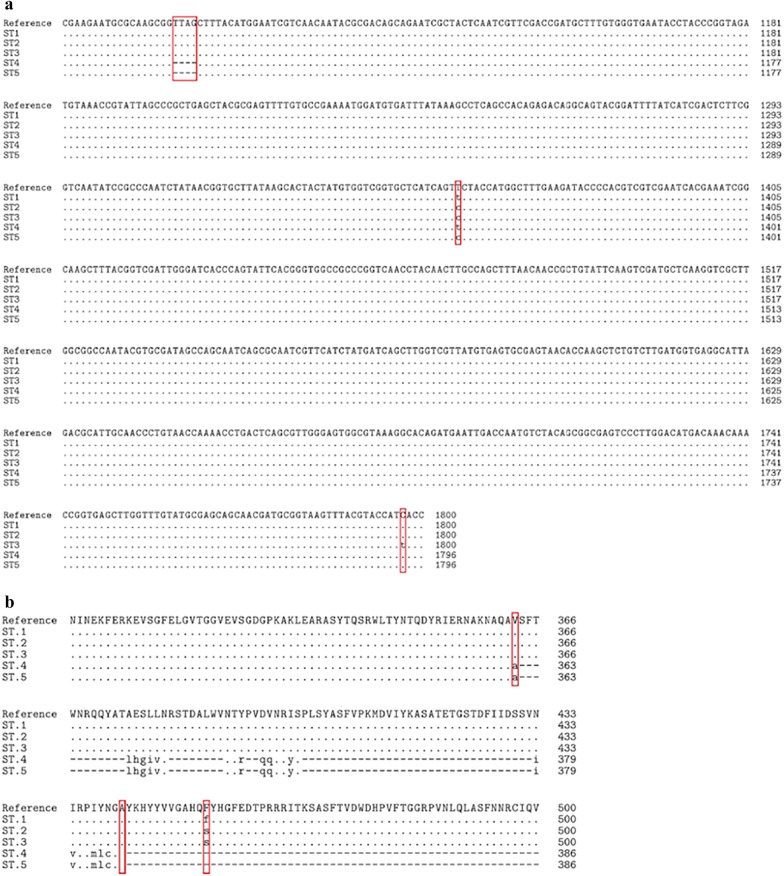
Alignment of the five *hlyA* nucleic acid sequences (**a**) and amino acid sequences (**b**). Variance with the reference sequence type (ST.1) is indicated by a red square

Furthermore, we randomly selected 85 toxigenic strains of O1 El Tor isolated from 1961 to 2007, and we performed hemolysis tests with these strains to confirm the hemolytic phenotype using strain C6706 as the positive control and CΔ*hlyA* as the negative control. Among them, 71 hemolytic and 14 nonhemolytic strains were identified. The *hlyA* genes of these strains were then amplified and sequenced. Using the *hlyA* sequence of strain C6706 as the reference, a total of five *hlyA* sequence types were found and designed as ST.1 to ST.5, respectively (Table 1 and Fig. 2). The sequences that were identical to C6706 *hlyA* were designated ST.1 in this study. ST. 2 had a base variance at 1358 within the *hlyA* ORF and resulted in 453S instead of 453F in ST.1. The sequence type ST.3 had the same base variance as ST.1, with an additional base variance at 1797 that was a synonymous mutation. ST.4 had a 4-base deletion mutation at positions 1088–1091, which caused the termination code to appear in the middle of the original ORF and the generation of a truncated and mutant protein during translation. ST.5 had the same 4-base deletion and the same mutation in ST.2 at position 1358. The sequence mutations and number of strains with hemolytic and non-hemolytic phenomena in each *hlyA* sequence type are listed in Table 1.

**Table 1:** The *hlyA* sequence types of the test strains and their hemolysis test results

Sequence types	Sequence variances	Amount of hemolytic strains	Amount of non-hemolytic strains
ST.1	Reference sequence type	25	12
ST.2	1358T → C, F → S	45	0
ST.3	1358T → C, F → S and 1797C → T (synonymous mutation)	1	0
ST.4	nt1088-1091 deletion	0	1
ST.5	nt1088-1091 deletion, 1358T → C, F → S	0	1

### Deletion of the *hlyA* base result in a hemolytic-negative strain

**Fig. 3:**
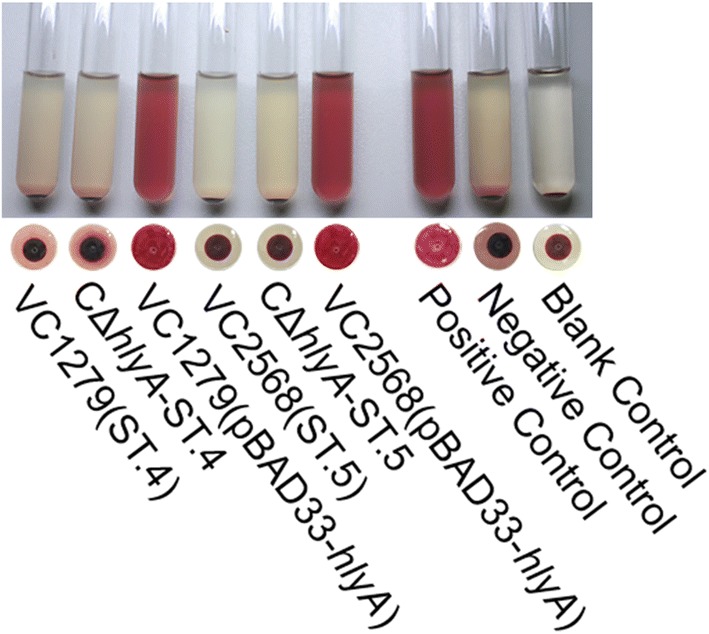
ST.4/ST.5 genes have no role in conferring hemolysis ability to the strains. CΔ*hlyA*-CST.4, the strain complemented with pBAD33-ST.4 into CΔ*hlyA*, remained non-hemolytic, whereas when VC1279 (ST.4 type of *hlyA*) and VC2568 (ST.5 type of *hlyA*) were complemented with pBAD33-*hlyA* to generate strain VC1279(pBAD33-*hlyA*) and VC2568(pBAD33-*hlyA*) respectively, their hemolysis tests became positive. The positive control was strain C6706, and the negative control was strain CΔ*hlyA*. The 1% sheep erythrocyte LB solution was used as the blank control

It was noticed that all ST.2 strains were hemolytic, although an amino acid variation occurred when compared with the reference sequence ST.1, showing that the F453S variation did not affect the hemolytic activity of HlyA. In fact, in another study [19], ST.2 type *hlyA* was considered a wild sequence type, and this type of strain was also hemolytic. In the *hlyA* sequence type ST.3, in addition to F453S variation, another point mutation at 1979 (C → T) occurred but was a synonymous mutation. This strain was also hemolytic. Both strains (VC1279 and VC2568) in ST.4 and ST.5 were hemolysis negative, presenting a 4-base deletion of nucleotide 1088 to 1091 in the *hlyA* ORF. In addition, a point mutation was detected at nucleotide position 1358, causing the F453S mutation in strain VC2568, such as ST.2, but it did not affect the hemolytic activity of HlyA. Using the genome DNAs of strains VC1279 and VC2568 as templates, we cloned their *hlyA* genes into the expression plasmid pBAD33, and we generated the recombinant plasmids pBAD33-ST.4 and pBAD33-ST.5 and transferred them into strain CΔ*hlyA,* respectively. Hemolysis tests showed that both complemental strains were still hemolytic-negative (Fig. 3). The recombinant plasmid pBAD33-hlyA was then transferred into strain VC1279 and VC2568, restoring the hemolytic phenotype (Fig. 3), which showed that the negative hemolytic phenotype resulted from the base deletion mutation in their *hlyA* genes.

### HlyA secretion deficiency or low expression was observed in some nonhemolytic strains of the ST.1 group

**Fig. 4:**
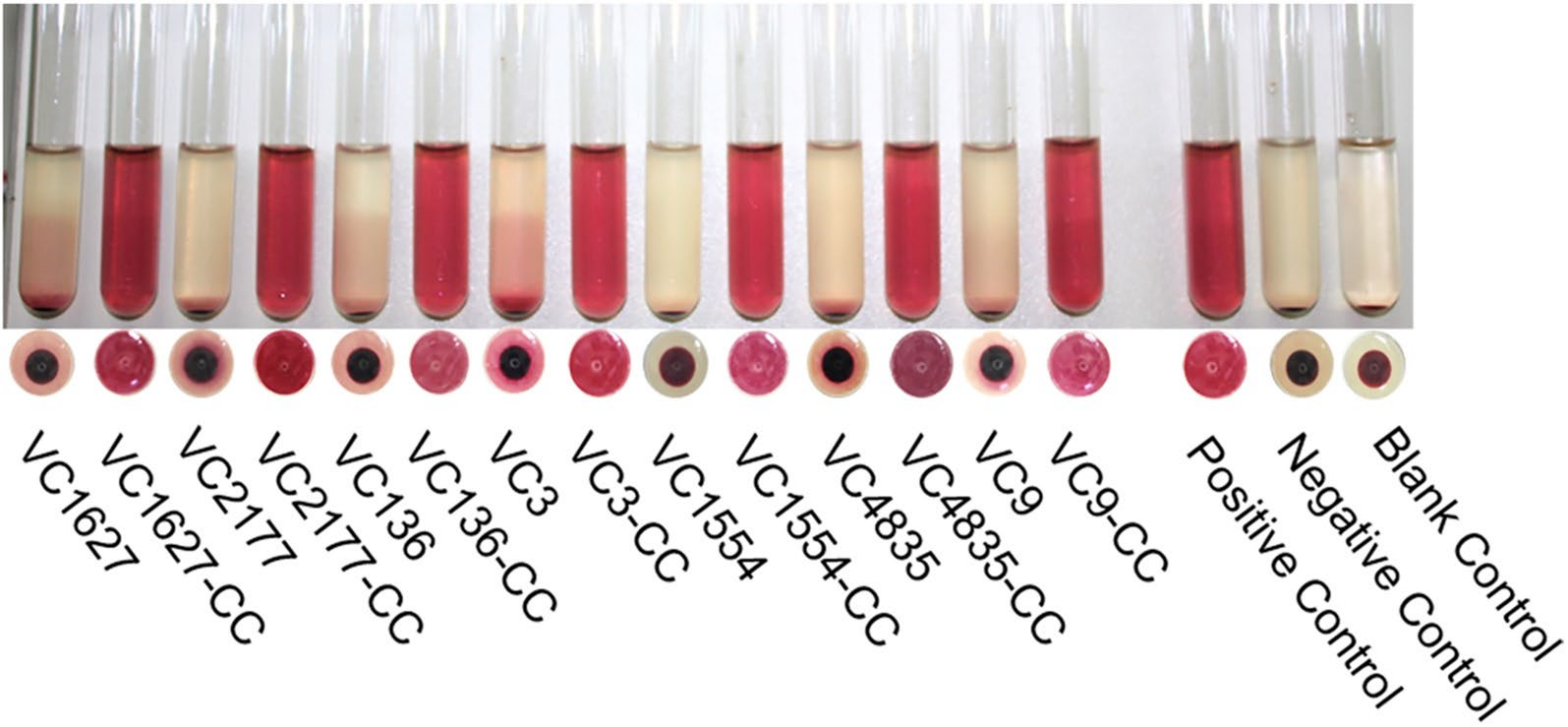
Hemolysis tests of the wildtype nonhemolytic strains and their cytoplasm samples. A strain name with “-CC” indicates the cytoplasm of this strain. Strain C6706 was used as the positive control, and strain CΔ*hlyA* was the negative control. The 1% sheep erythrocyte LB solution was used as the blank control

Among the 37 strains with the ST.1 sequence type of *hlyA*, the hemolysis tests showed that 25 strains were hemolytic, but 12 were non-hemolytic. We then focused on these hemolytic-negative strains of ST.1 (ST.1^H−^ strains). HlyA is secreted extracellularly through T1SS in *V. cholerae*. The secretion of HlyA was detected by measuring the sheep blood cell hemolysis in the supernatant and cytoplasm of bacterial cells. Liquid cultures of all 12 ST.1^H−^ strains with an OD_600_ of 0.6 were washed and disrupted by ultrasound, and the supernatants of the solution were obtained for the hemolysis tests, respectively. The positive control strain C6706 and negative control strain CΔ*hlyA* were treated using the same protocol. Among the 12 ST.1^H−^ strains, seven showed positive hemolysis (Table 2 and Fig. 4), suggesting that in these seven strains, HlyA could be expressed but not secreted normally, resulting in hemolytic activity in the cytoplasm but non-hemolytic activity when tested with the cultured bacteria.

**Table 2:** Hemolysis and transcription analysis of the non-hemolytic El Tor strains (ST.1^H−^) in ST.1 group

Strains	Wildtype strains		Strains carrying pBAD33-*hlyA*		*hlyA* transcription analysis
	Hemolysis test	Cytoplasmic hemolysis test	Hemolysis test	Cytoplasmic hemolysis test	
VC1627	–	+	+	ND	0.024
VC1554	–	+	+	ND	0.015
VC9	–	+	+	ND	ND
VC3	–	+	–	+	1.284
VC136	–	+	–	+	1.132
VC2177	–	+	–	+	5.856
VC4835	–	+	–	+	2.411
VC1301	–	–	+	ND	0.228
VC4979	–	–	+	ND	0.305
VC4826	–	–	+	ND	0.318
VC4983	–	–	+	ND	ND
VC4732	–	–	+	ND	ND

*ND* Not selected and determined

**Fig. 5:**
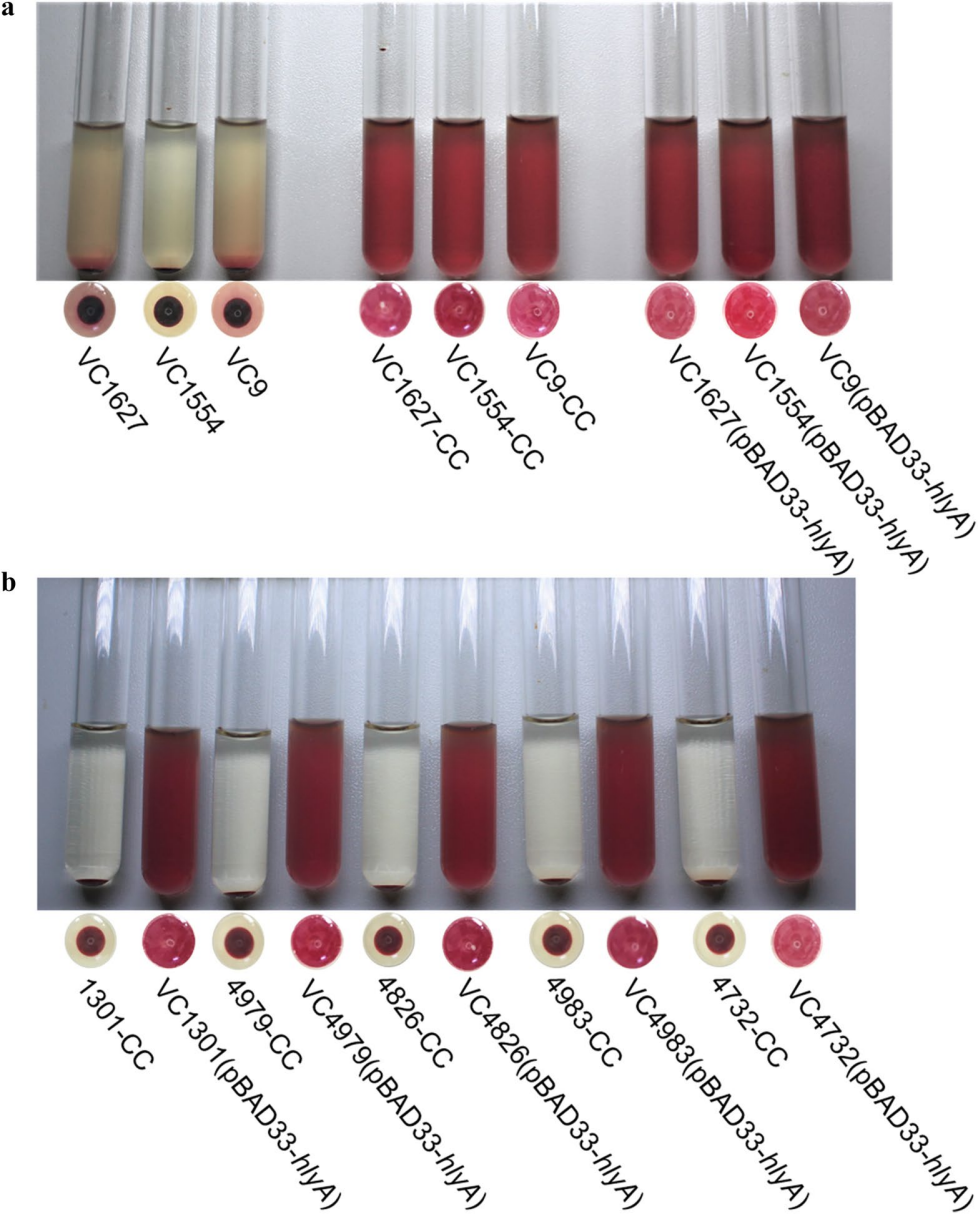
Hemolysis tests for eight ST.1^H−^ strains complemented with the *hlyA* overexpression plasmid pBAD33-*hlyA*. A strain name with “-CC” indicates the cytoplasm of this strain. **a** Hemolysis results for strains VC1627, VC1554 and VC9 and their cytoplasm, and the corresponding complemented strains with pBAD33-*hlyA* (strain code with “(pBAD33-*hlyA*)”). **b** Hemolysis test results for the strain group containing VC1301, VC4979, VC4826 and VC4732, showing hemolysis of their cytoplasm and the corresponding strains complemented with pBAD33-*hlyA*

In parallel, hemolysis of these ST.1^H−^ strains was also tested by increasing the expression of *hlyA* genes through the introduction of plasmid pBAD33-*hlyA* into these strains. Eight strains became hemolytic in the hemolysis tests using the cultured bacterial cells (Table 2 and Fig. 5). Among them, three strains (VC1627, VC1554 and VC9) were previously non-hemolytic, but their cytoplasm and their *hlyA* overexpression transformants were hemolytic (Table 2 and Fig. 5a), suggesting that, for these wildtype strains, HlyA expression was too low, with normal or less efficient HlyA secretion. Five strains (VC1301, VC4979, VC4826, VC4983 and VC4732) for which the cytoplasm was still non-hemolytic became hemolytic when transformed with the *hlyA* overexpression plasmids (Table 2 and Fig. 5b), which might suggest that their *hlyA* genes were expressed at low levels in the cytoplasma, but the secretion of HlyA was normal.

**Fig. 6:**
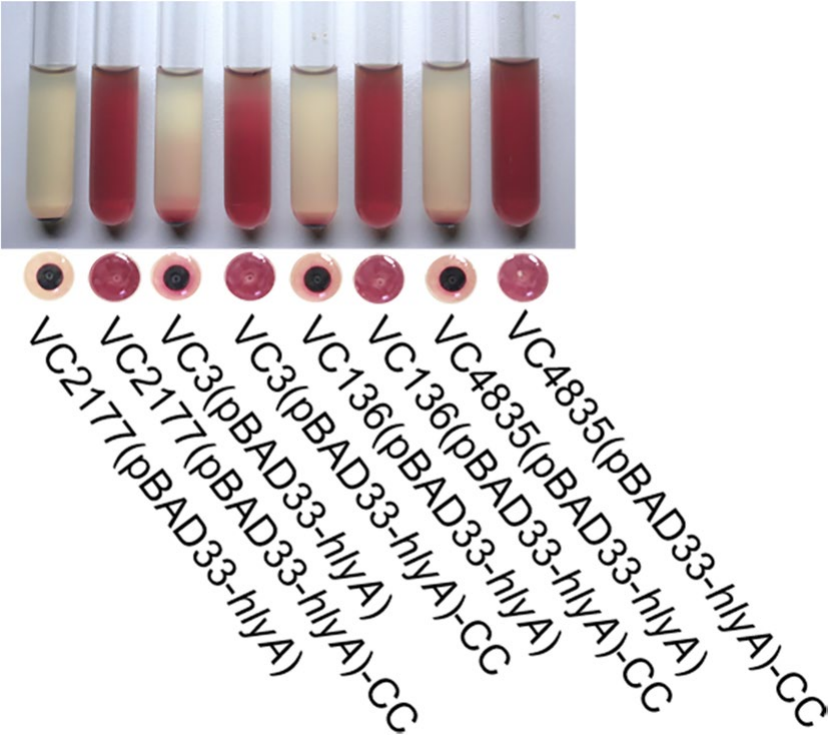
Hemolysis tests of four ST.1^H−^ strains (VC2177, VC3, VC136 and VC4835) complemented with pBAD33-*hlyA* and their cytoplasm. The codes of four wildtype strains with “(pBAD33-*hlyA*)” indicate the complemented strains with pBAD33-*hlyA* with increased expression of HlyA. A strain code with “-CC” indicates the cytoplasm of this strain

Four ST.1^H−^ strains (VC3, VC136, VC2177 and VC4835) remained non-hemolytic when they were transferred with plasmid pBAD33-*hlyA* and tested with the cultured cells (Table 2 and Fig. 6). These strains were ultrasonically disrupted, and the hemolysis of their cytoplasm was tested. All of them were hemolytic, strongly suggesting an abnormal HlyA secretion; however, their *hlyA* transcription and expression might have been normal, since without the introduction of pBAD33-*hlyA*, the cytoplasm still possessed hemolytic activities.

We further determined the transcription levels of the *hlyA* gene in these four ST.1^H−^ wildtype strains (VC3, VC136, VC2177 and VC4835), using the *recA* gene of C6706 as the internal reference. The transcription levels of *hlyA* in this group were relatively high (Table 2). The *hlyA* transcription levels of the five ST.1^H−^ wildtype strains (VC1301, VC4979, VC4826, VC4983 and VC4732) were also determined, and they demonstrated much lower *hlyA* transcription levels than the previous group (Table 2). These data support that, in these five strains, expression of HlyA was reduced, but there was no deficiency in HlyA secretion. In contrast, four strains (VC3, VC136, VC2177 and VC4835) had normal expression but blocked secretion of HlyA.

## Discussion

Hemolysis is related to pathogenicity in some pathogenic bacteria, and it is also used for the biological classification or description of biological characteristics in some bacteria. In this study, we tested and analyzed the non-hemolytic *V. cholerae* El Tor strains of the seventh pandemic as well as possible factors affecting the hemolytic activity of HlyA. We found that in addition to *hlyA* gene mutations, transcription/expression and blockade of the secretion of this gene and product may also be involved in nonhemolysis of the strains.

In the sixth pandemic, the classical biotype of serogroup O1 *V. cholerae* did not show hemolytic activity toward sheep erythrocytes. In the early stage of the seventh pandemic, the El Tor strain had hemolytic activity, but later the nonhemolytic *V. cholerae* emerged, and thus the hemolysis test could not be used as an indicator to distinguish the two biological types. The emergence of nonhemolytic strains may indicate the genetic variation of El Tor strains. HlyA is the main factor used by *V. cholerae* to lyse sheep erythrocytes. In the 85 O1 El Tor strains isolated in different years selected for this study, all of them carried the *hlyA* gene, but five variant sequence types of the gene were found, showing mutations of this gene during the transmission of El Tor biotype *V. cholerae* in the seventh pandemic. It was further found that some mutations in the *hlyA* gene might not affect its hemolytic activity toward sheep erythrocytes, but a 4-base deletion in *hlyA* was found to be responsible for the loss of hemolysis by the strains; this deletion leads to the early appearance of the TGA termination signal and will result in the expression of a truncated protein. This is one of the mechanisms leading to the nonhemolysis variation of *V. cholerae*.

Positive and negative hemolytic phenotypes appeared in the strains with the same *hlyA* sequence as hemolytic strain C6706, suggesting the presence of a complex non-hemolytic mechanism in these strains. In the *hlyA*-mediated hemolysis process, the first step was the expression of HlyA, followed by the secretion of HlyA out of bacterial cells. We found that some strains with intact *hlyA* genes were non-hemolytic in the hemolysis tests, but their *hlyA* genes demonstrated a high transcription level and their cytoplasm had hemolytic activity. Introduction of the *hlyA* overexpression plasmids was still unable to transform the strains into hemolytic strains in the test. These data strongly suggested that HlyA secretion was blocked in these strains.

For the other non-hemolytic strains with intact *hlyA* genes, a lower level of *hlyA* gene transcription was found in these wildtype strains. These findings were supported by the direct *hlyA* gene transcription analysis and/or experiments showing that increasing HlyA expression in the cells resulted in the appearance of hemolytic activity in the hemolysis tests with cultured bacteria; however, the secretion of HlyA in these strains should be normal, or at least not obviously affected. For such strains, it can be concluded that their non-hemolytic phenotype resulted from low-level transcription of the *hlyA* gene.

## Conclusions

Based on the above phenotypic, subcellular and gene transcription studies, the possible mechanisms causing the non-hemolytic phenotypes of the *V. cholerae* El Tor strains carrying intact *hlyA* genes were observed: (1) The base deletion in the *hlyA* gene leads to a frameshift mutation and generates an abnormal HlyA protein and a loss of hemolytic activity. (2) The *hlyA* gene is not expressed in bacteria or has a low expression level and low activity. (3) HlyA expression is normal, but its secretion is blocked and causes a non-hemolytic phenotype of the strain in the hemolytic test. It is noteworthy that the transcription level of the *hlyA* gene cannot be used as an indicator for the hemolytic activity of *V. cholerae*. Our study also showed that the regulation of the hemolytic phenotype of *V. cholerae* is very complex. In addition to the main role of *hlyA*, other genes such as *hlyB*, *hlyD*, and *tolC* are also involved in the expression and secretion of HlyA [20]. Moreover, the quorum-sensing system and its regulatory proteins HapR, Fur and HlyU regulate the expression of *hlyA* together in *V. cholerae* [21]. Therefore, our study may provide future analysis points regarding the transcriptional regulation of the *hlyA* gene and HlyA secretory system, to reveal abnormal changes in these regulatory parameters and secretory processes in non-hemolytic strains.

## Materials and methods

### Bacterial strains, culture conditions and plasmids

The wildtype (WT) serogroup O1, El Tor biotype *V. cholerae* C6706 and its derivative mutants were grown in Luria–Bertani (LB) broth (pH 7.4) containing 1% NaCl (170 mM) at 37 °C unless specifically indicated. *E. coli* DH5α*λpir* and SM10*λpir* were cultured at 37 °C and used for cloning purposes. The culture media were supplemented with ampicillin (Amp, 100 mg/ml), streptomycin (Sm, 100 mg/ml) or chloramphenicol (Cm, 10 mg/ml) as required. All strains and plasmids used in this study are listed in Table 3.

**Table 3:** Strains and plasmids used in this study

Strains/plasmids	Characters	Reference
*V. cholerae*		
C6706	El Tor biotype, serogroup O1, Sm^r^	Lab collections
CΔ*hlyA*	*hlyA* deletion of C6706, Sm^r^	This study
CΔ*hlyA*-C	CΔ*hlyA* strains was complemented with pBAD33-*hlyA*	This study
CΔ*hlyA*-CST.4	CΔ*hlyA* strains was complemented with pBAD33-ST.4	This study
CΔ*hlyA*-CST.5	CΔ*hlyA* strains was complemented with pBAD33-ST.5	This study
CΔ*hlyA*-CK	CΔ*hlyA* strains was complemented with pBAD33 for control	This study
*E. coli*		
SM10*λpir*	Km*, thi thr leu tonA lacY supE recA::RP4*-*2*-*TC::Mu* λ*pir*	Lab collections
DH5α*λpir*	*supE44* Δ*lacU169 (ΦlacZ*Δ*M15) recA1 endA1 hsdR17 thi*-*1 gyrA96 relA1 λpir*	Lab collections
Plasmids		
pWM91	Suicide plasmid; *oriR oriT lacZ tetAR sacB*, AMP^r^	Lab collections
pWM91-Δ*hlyA*	pWM91 carrying upstream and downstream fragments flanking *hlyA*	This study
pBAD33	*E. coli* expression vector, Cm^r^	Lab collections
pBAD33-*hlyA*	pBAD33 containing *hlyA*, Cm^r^	This study
pBAD33-ST.4	pBAD33 containing ST.4 *hlyA*, Cm^r^	This study
pBAD33-ST.5	pBAD33 containing ST.5 *hlyA*, Cm^r^	This study

In this study, 85 strains of O1 El Tor toxigenic strains isolated from different years (1961–2007) in China were randomly selected from the National *Vibrio* Collection Laboratory in National Institute for Communicable Disease Control and Prevention, Chinese Center for Disease Control and Prevention (China CDC), which is operated in our laboratory. Biotype identification of the serogroup O1 strains is performed in the local public health laboratories of the CDCs in different provinces before their submissions to the Collection Laboratory, and the strains were selected randomly for the biotype confirmation in this laboratory. The following criteria for biotype identification are conducted in all the public health laboratories in CDCs: the lysis by bacteriophage of classical IV, polymyxin B sensitivity and agglutination of chicken erythrocytes. The year of isolation and reconfirmed hemolytic phenotype of the strain in this study are shown in Additional file 1: Table S1.

### Construction of mutants

The *V. cholerae* deletion mutant was constructed with the reference strain C6706 by allelic gene exchange. Primers were designed based on the genome sequence of *V. cholerae* strain C6706. All primers used in this study were designed with SnapGene (GSL Biotech) and then blasted in primer-BLAST of PubMed (https://www.ncbi.nlm.nih.gov/tools/primer-blast/). To construct the *hlyA* gene deletion mutant of CΔ*hlyA* from strain C6706, the upstream and downstream DNA fragments flanking the *hlyA* open reading frame (ORF) were amplified from C6706 genomic DNA using primer pairs *hlyA*-*Xho*I-F/middle-R and middle-F/*hlyA*-*Spe*I-R, respectively. The amplicons were mixed in equimolar concentrations and used as template to amplify the chromosomal fragment containing the *hlyA* deletion using the primer pair *hlyA*-*Xho*I-F/*hlyA*-*Spe*I-R. The resulting fragment was cloned into the suicide plasmid pWM91 to generate pWM91-Δ*hlyA.* pWM91-*hlyA* was constructed in *E. coli* DH5α*λpir*. pWM91-*hlyA* was extracted from DH5α*λpir*, transformed into strain SM10*λpir*, and mixed with strain C6706 for conjugation of pWM91-*hlyA*. Exconjugants were selected in LB medium containing Amp and Sm, and they were streaked on LB agar containing 15% (w/v) sucrose. Sucrose-resistant colonies were selected and tested for Amp sensitivity, and the *hlyA* deletion mutant of *V. cholerae* was confirmed by DNA sequencing.

To complement the tested *hlyA* genes in the *hlyA* mutants of CΔ*hlyA*, plasmid pBAD33 was used to construct complementary plasmids carrying the inserted wildtype *hlyA* or its derivatives. The fragment containing *hlyA* was PCR-amplified from chromosomal DNA of C6706 with primers *hlyA*-*Xba*I-F/*hlyA*-*Kpn*I-R, digested with the restriction enzymes *Xba*I/*Kpn*I and inserted into pBAD33. The complementary plasmids were transformed into CΔ*hlyA* or other mutants and induced with 0.01% arabinose for pBAD33. pBAD33 was also transformed into CΔ*hlyA* as the negative control. The complemented strains were verified by hemolysis tests. All primers used in this study are listed in Additional file 1: Table S2.

### Quantitative reverse transcription PCR (qRT-PCR)

*Vibrio cholerae* strains were grown in LB medium to an OD_600_ of 0.6. Total RNA was extracted from the culture of C6706 and other test strains using the SuperScript™ III Reverse Transcriptase and DNA-free™ DNA Removal Kit (Thermo Fisher). The RNA samples were analyzed by quantitative real-time reverse transcription-PCR (qRT-PCR) using the One Step SYBR Primerscript RT-PCR Kit II (TaKaRa). Relative expression values (R) were calculated using the equation ${\text{R}} = 2^{{ - \left( {\Delta Cq target - \Delta Cq reference} \right)}}$, where Cq is the fractional threshold cycle. *recA* of C6706 was used as an internal reference. Run the qRT-PCR as follows: Pre-incubation (1 cycle): 95 °C for 1 min; Amplification (40 cycles): Denaturation at 95 °C for 10 s, Annealing at 60 °C for 30 s (to collect fluorescence signals); Melting (1 cycle): 95 °C for 10 s, 65 °C for 30 s, and 97 °C for 1 s; Cooling (1 cycle): 37 °C for 30 s. A control mixture using total RNA as a template was performed for each reaction to exclude chromosomal DNA contamination. The primers used for these target genes, *recA* and *hlyA* are listed in Additional file 1: Table S2.

### Ultrasonic breaking of culture bacterial cells

*Vibrio cholerae* strains were cultured in LB to an OD_600_ of 0.6, and 20 ml liquid culture was centrifuged at 5000 r/min. The supernatant was discarded, and the cells were resuspended in 20 ml LB and then centrifuged for washing two times. For ultrasonic cell disruption, the sample tube of bacteria was placed in an ice bath. The ultrasonic breaking procedure was set as ultrasound for 5 s, with 5-s intervals, for a total of 5 min. After ultrasonic disruption, the sample cytoplasm was collected by centrifugation at 12,000 r/min and 4 °C, followed by resuspension in 20 ml LB.

### Hemolysis test of *V. cholerae* strains and cytoplasm

Sheep blood was washed twice with sterilized isotonic sodium chloride solution at a threefold volume. During the third wash, the mixture was centrifuged at 2000 r/min for 10 min, and the sheep erythrocytes were obtained by discarding the supernatant of the mixture. Then, 1% sheep erythrocyte solution was prepared with sterilized isotonic sodium chloride solution, and 1 ml bacterial culture (OD_600_ of 0.6) and 1 ml of 1% sheep erythrocyte solution were mixed in a test tube, incubated at 37 °C for 2 h in bacteriology incubator, and left at 4 °C overnight. Hemolysis was estimated by comparison with the positive and negative controls. For the hemolysis assay of the bacterial cytoplasm, the culture of the strain with an OD_600_ of 0.6 was washed twice with sterilized isotonic sodium chlorides solution, resuspended in the same volume as the previous medium, and disrupted using the ultrasonic technique as described above.

## Additional file

**Additional file 1: Table S1.** Hemolysis and isolation years of the wildtype *V. cholerae* El Tor strains used in this study. **Table S2.** Primers used in this study.

## Data Availability

The datasets during and/or analysed during the current study available from the corresponding author on reasonable request.
