# Supplementary material for: Nonhemolysis of epidemic El Tor biotype strains of Vibrio cholerae is related to multiple functional deficiencies of hemolysin A
Source: Gut Pathog. 2019 Jul 12;11:38. doi: 10.1186/s13099-019-0316-7 (PMC6626427; doi:10.1186/s13099-019-0316-7)
Supplement: Supplementary file 1 — Additional file 1: Table S1. Hemolysis and isolation years of the wildtype V. cholerae El Tor strains used in this study. Table S2. Primers used in this study. [file 13099_2019_316_MOESM1_ESM.docx]

**Additional file Tables**

**Table S1.** Hemolysis and isolation years of the wildtype *V. cholerae* El Tor strains used in this study.

| Strains | Isolation year | Hemolysis |  | Strains | Isolation year | Hemolysis | |
| --- | --- | --- | --- | --- | --- | --- | --- |
| VC2541 | 1962 | + |  | VC2870 | 1961 | **+** |  |
| VC2542 | 1962 | + |  | VC2871 | 1961 | **+** |  |
| VC2544 | 1962 | + |  | VC2872 | 1961 | **+** |  |
| VC2545 | 1962 | + |  | VC2873 | 1961 | **+** |  |
| VC2546 | 1962 | + |  | VC2880 | 1961 | **+** |  |
| VC2547 | 1962 | + |  | VC2882 | 1961 | **+** |  |
| VC2548 | 1962 | + |  | VC2883 | 1961 | **+** |  |
| VC2549 | 1962 | + |  | VC2886 | 1961 | **+** |  |
| VC2550 | 1962 | + |  | VC2888 | 1961 | **+** |  |
| VC2551 | 1963 | + |  | VC2891 | 1961 | **+** |  |
| VC2552 | 1963 | + |  | VC2892 | 1961 | **+** |  |
| VC2553 | 1963 | + |  | VC2893 | 1961 | **+** |  |
| VC2555 | 1963 | + |  | VC2895 | 1961 | **+** |  |
| VC2558 | 1963 | + |  | VC2896 | 1961 | **+** |  |
| VC2559 | 1963 | + |  | VC2897 | 1961 | **+** |  |
| VC2560 | 1963 | + |  | VC2915 | 1962 | **+** |  |
| VC2561 | 1963 | + |  | VC2916 | 1962 | **+** |  |
| VC2562 | 1963 | + |  | VC2917 | 1962 | **+** |  |
| VC2866 | 1961 | + |  | VC2919 | 1962 | **+** |  |
| VC2921 | 1962 | + |  | VC2920 | 1962 | **+** |  |
| VC2923 | 1962 | + |  | VC4732 | 1994 | **-** |  |
| VC5537 | 1963 | + |  | VC5039 | 1994 | **+** |  |
| VC46 | 2005 | + |  | VC5427 | 1996 | **+** |  |
| VC929 | 1995 | + |  | VC5735 | 1995 | **+** |  |
| VC4412 | 1961 | + |  | VC136 | 2001 | **-** |  |
| VC2742 | 1961 | + |  | VC137 | 2001 | **+** |  |
| VC2743 | 1961 | + |  | VC3 | 1986 | **-** |  |
| VC2744 | 1962 | + |  | VC1279 | 1979 | **-** |  |
| VC2813 | 1990 | + |  | VC1283 | 1984 | **+** |  |
| VC936 | 1998 | + |  | VC1295 | 1988 | **+** |  |
| VC955 | 2001 | + |  | VC1547 | 1978 | **+** |  |
| VC1017 | 2000 | + |  | VC1552 | 1979 | **+** |  |
| VC1301 | 1998 | - |  | VC1553 | 1980 | **+** |  |
| VC1592 | 1992 | + |  | VC1554 | 1981 | **-** |  |
| VC1627 | 1997 | - |  | VC2568 | 1978 | **-** |  |
| VC2177 | 1995 | - |  | VC2578 | 1985 | **+** |  |
| VC2643 | 1993 | + |  | VC4826 | 1980 | **-** |  |
| VC2645 | 1995 | + |  | VC4828 | 1981 | **+** |  |
| VC4723 | 1991 | + |  | VC4835 | 1982 | **-** |  |
| VC9 | 2001 | - |  | VC4979 | 1979 | **-** |  |
| VC10 | 2005 | + |  | VC4983 | 1985 | **-** |  |
| VC5776 | 2007 | + |  | VC5041 | 1984 | **+** |  |
| VC5777 | 2007 | + |  |  |  |  |  |

**Table S2.** Primers used in this study.

| Primer Name | Primer sequence （5’→3’） | Tm  (°C) | molecular weight (g/mol) |
| --- | --- | --- | --- |
| *hlyA*-XhoI-F | ccgCTCGAGCTGTGATCCGCTGTGAATTTTC | 70.8 | 9462 |
| *hlyA*-SpeI-R | cggACTAGTGATACCGACACGCATCCGAGGAATACG | 74 | 11079 |
| middle-F | CTAAAAATAACAGAGTCAGTGAGGTTTATAAAGAGACCGTCAACATAGCCATTAAAC | 72.2 | 17593 |
| middle-R | GTTTAATGGCTATGTTGACGGTCTCTTTATAAACCTCACTGACTCTGTTATTTTTAG | 72.2 | 17476 |
| *hlyA*-F | AGCCAAACTAGAGGCGAGA | 58 | 5875 |
| *hlyA*-R | CACAAAACTCGCGTAGCTCA | 60 | 6053 |
| *hlyA*-XbaI-F | cggGGTACCaggaggaattcaccATGCCAAAACTCAATCG | 74.5 | 12299 |
| *hlyA*-KpnI-R | TGCTCTAGATTAGTTCAAATCAAATTGAACC | 61.5 | 9474 |
| *racA*-F | GTGCTGTGGATGTCATCGTTGTTG | 57 | 7429 |
| *racA*-R | CCACCACTTCTTCGCCTTCTTTGA | 57 | 7165 |

^a^Restriction sites are underlined.
